# [1-(5-Bromo-2-oxidobenzyl­idene)thio­semicarbazidato-κ^3^ *O*,*N* ^1^,*S*](pyridine-κ*N*)nickel(II)

**DOI:** 10.1107/S1600536812028917

**Published:** 2012-08-01

**Authors:** Fernanda Rosi Soares Pederzolli, Leandro Bresolin, Johannes Beck, Jörg Daniels, Adriano Bof de Oliveira

**Affiliations:** a Escola de Química e Alimentos, Universidade Federal do Rio Grande, Av. Itália km 08, Campus Carreiros, 96203-900 Rio Grande, RS, Brazil; b Institut für Anorganische Chemie, Universität Bonn, Gerhard-Domagk-Strasse 1, D-53121 Bonn, Germany; c Departamento de Química, Universidade Federal de Sergipe, Av. Marechal Rondon s/n, Campus, 49100-000 São Cristóvão, SE, Brazil

## Abstract

The reaction of 5-bromo­salicyl­aldehyde thio­semicarbazone with nickel acetate tetra­hydrate and pyridine yielded the title compound, [Ni(C_8_H_6_BrN_3_OS)(C_5_H_5_N)]. The Ni^II^ atom is four-coordinated in a square-planar environment by one deprotonated dianionic thio­semicarbazone ligand, acting in a tridentate chelating mode through N, O and S atoms forming two metalla-rings, and by one pyridine mol­ecule. The complex mol­ecules are linked into dimers by pairs of centrosym­metrical N—H⋯N inter­actions. In addition, mol­ecules are connected through inter­molecular Br⋯Br inter­actions [3.545 (1) Å], forming chains along the *b*-axis direction.

## Related literature

For the synthesis of 5-bromo­salicyl­aldehyde thio­semi­carba­zones and for the anti­bacterial activity of their complexes, see: Joseph *et al.* (2010 ▶). For the crystal structure of 5-bromo­salicyl­aldehyde thio­semicarbazone, see: Kargar *et al.* (2010 ▶). For the crystal structure of an Ni^II^ complex with a similar coordination environment, see: Güveli *et al.* (2009 ▶). For the coordination chemistry of thio­semicarbazone derivatives, see: Lobana *et al.* (2009 ▶).
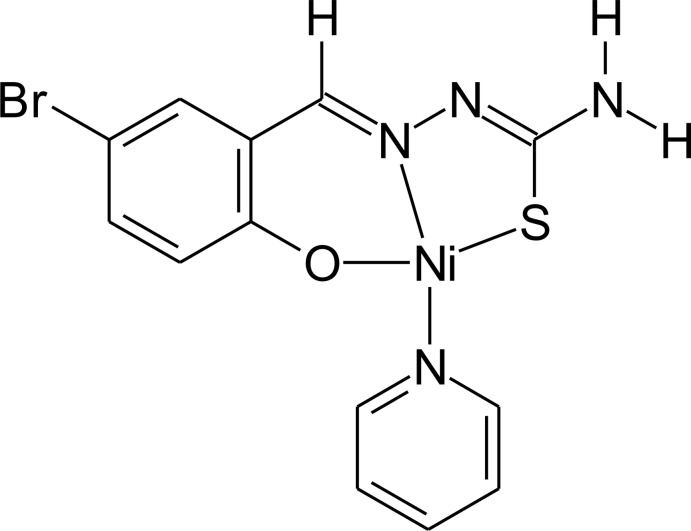


## Experimental

### 

#### Crystal data

[Ni(C_8_H_6_BrN_3_OS)(C_5_H_5_N)]
*M* *_r_* = 409.94
Monoclinic, 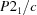

*a* = 12.2447 (4) Å
*b* = 4.1135 (1) Å
*c* = 31.1380 (11) Å
β = 112.646 (1)°
*V* = 1447.46 (8) Å^3^
*Z* = 4
Mo *K*α radiation
μ = 4.25 mm^−1^
*T* = 293 K
0.93 × 0.10 × 0.05 mm

#### Data collection

Nonius KappaCCD diffractometer
Absorption correction: multi-scan (Blessing, 1995 ▶) *T*_min_ = 0.443, *T*_max_ = 0.830
13946 measured reflections
3224 independent reflections
2697 reflections with *I* > 2σ(*I*)
*R*_int_ = 0.051

#### Refinement

*R*[*F* ^2^ > 2σ(*F* ^2^)] = 0.031
*wR*(*F* ^2^) = 0.083
*S* = 1.05
3224 reflections
190 parameters
H-atom parameters constrained
Δρ_max_ = 0.61 e Å^−3^
Δρ_min_ = −0.66 e Å^−3^

### 

Data collection: *COLLECT* (Nonius, 1998 ▶); cell refinement: *SCALEPACK* (Otwinowski & Minor, 1997 ▶); data reduction: *DENZO* (Otwinowski & Minor, 1997 ▶) and *SCALEPACK*; program(s) used to solve structure: *SHELXS97* (Sheldrick, 2008 ▶); program(s) used to refine structure: *SHELXL97* (Sheldrick, 2008 ▶); molecular graphics: *DIAMOND* (Brandenburg, 2006 ▶); software used to prepare material for publication: *publCIF* (Westrip, 2010 ▶).

## Supplementary Material

Crystal structure: contains datablock(s) I, global. DOI: 10.1107/S1600536812028917/zl2486sup1.cif

Structure factors: contains datablock(s) I. DOI: 10.1107/S1600536812028917/zl2486Isup2.hkl

Additional supplementary materials:  crystallographic information; 3D view; checkCIF report

## Figures and Tables

**Table 1:** Hydrogen-bond geometry (Å, °)

*D*—H⋯*A*	*D*—H	H⋯*A*	*D*⋯*A*	*D*—H⋯*A*
N3—H1⋯N2^i^	0.78	2.31	3.095 (3)	178

Symmetry code: (i) 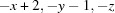.

**Fig. 1.**
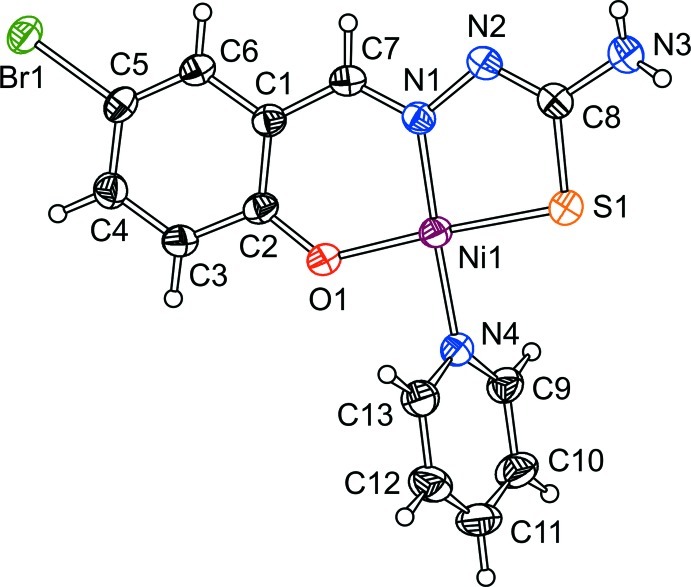
: The molecular structure of the title compound with labeling and displacement ellipsoids drawn at the 40% probability level.

**Fig. 2.**
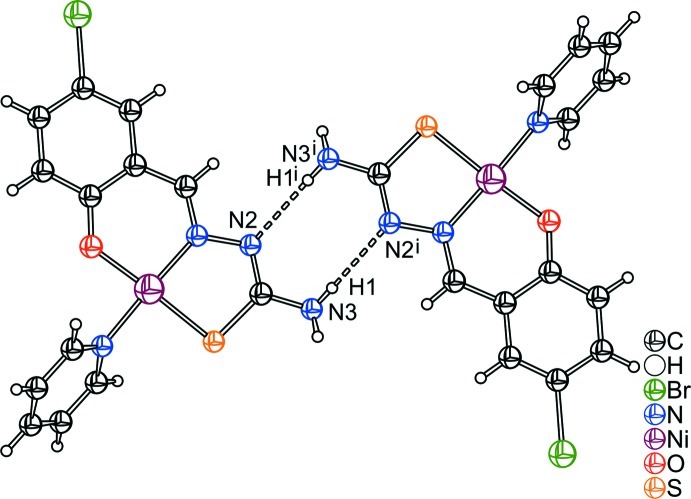
: Molecules of the title compound connected through pairs of inversion symmetric N—H···N interactions. Hydrogen bonding is indicated by dashed lines. Symmetry code: (i) -x, -y + 1, -z.

**Fig. 3.**
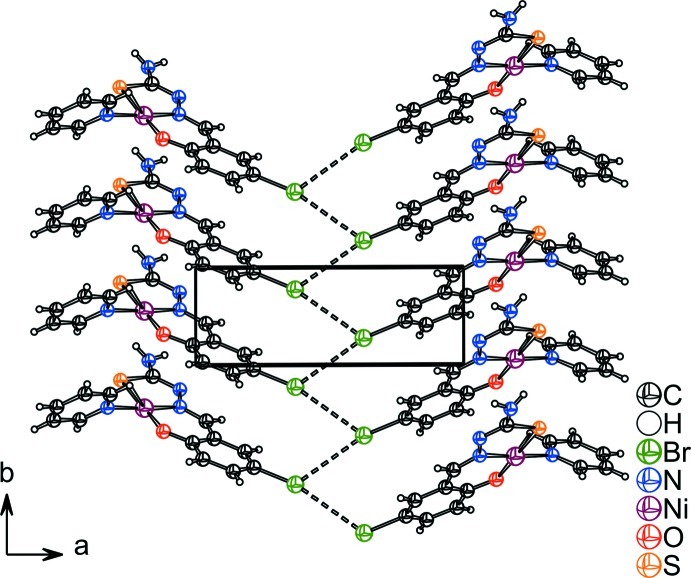
: Molecules of the title compound connected through intermolecular Br···Br interactions into chains along the crystallographic b direction. The Br···Br distances amount to 3.545 (1) Å and the interactions are indicated by dashed lines.

## supplementary crystallographic information

### Comment

Thiosemicarbazone derivatives have a wide range of applications in biological inorganic chemistry and a very interesting coordination chemistry (Lobana *et al.*, 2009). For example, Cu^II^ and Ni^II^ complexes with 5-bromosalicylaldehyde thiosemicarbazone show antibacterial activity against *Staphylococcus aureus* and *Escherichia coli* (Joseph *et al.*, 2010). As part of our study of thiosemicarbazone derivatives, we report herein the synthesis and the crystal structure of a new Ni^II^ complex with 5-bromosalicylaldehyde thiosemicarbazone. In the title compound, in which the molecular structure unit matches the asymmetric unit, the Ni^II^ ion is coordinated in a square planar environment by one deprotonated dianionic 5-bromosalicylaldehyde thiosemicarbazone and one pyridine ligand (Fig. 1). The selected bond angles formed between donor atoms trough the Ni atom are N1—Ni1—N4 = 177.00 (10)° and O1—Ni1—S1 = 176.46 (6)°, and show a slightly distorted coordination environment. The thiosemicarbazone ligand is coordinated to the Ni^II^ ion in a tridentate chelating mode, forming five- and six-membered rings, as a "NOS" donor with the O/S atoms *trans* to each other, while the N1 azomethine atom is *trans* to the N4 atom from the pyridine ligand.

The acidic hydrogen of the hydrazine fragment is lost by the reaction with KOH, which is in agreement with thiosemicarbazone derivatives prepared from aldehydes or ketones. The negative charge is delocalized over the C—N—N—C—S fragment as indicated by their intermediate bond distances. The imine and thioamide C—N distances indicate considerable double bond character, while the C—S distance is consistent with increased single bond character. These distances are C7—N1 = 1.295 (3) Å, N1—N2 = 1.403 (3) Å, N2—C8 = 1.289 (4) Å and C8—S1 = 1.735 (3) Å. The hydrogen of the hydroxyl group is also deprotonated with KOH, resulting in the dianionic form of the ligand.

The ligand shows a Z—E—E—*Z* conformation for the donor atoms about the C1—C7/C7—N1/N1—N2/N2—C8 bonds and the mean deviations from the least squares planes for the chelated fragments Ni1/N1/C7/C1/C2/O1 and Ni1/N1/N2/C8/S1 amount to 0.0286 (15) Å for N1 and 0.0170 (12) Å for N1, respectively, and the dihedral angle between the two planes is 2.97 (11)°. The Z—E—E—*Z* conformation is also observed for the free ligand (Kargar *et al.*, 2010) as well as for a complex with similar coordination environment (Güveli *et al.*, 2009).

Both ligands are almost planar (Fig. 1 and Fig. 2) and the maximum deviation from the least squares plane through all non-hydrogen atoms for the deprotonated thiosemicarbazone fragment C1/C2/C3/C4/C5/C6/C7/C8/Br1/N1/N2/N3/O1/S1 and for the pyridine molecule C9/C10/C11/C12/C13/N4 amount to 0.0668 (25) Å for C7 and 0.0059 (21) Å for C9, respectively, and the dihedral angle between the two planes is 61.15 (6)°.

The molecules are linked by pairs of centrosymmetrical N—H···N interactions (Fig. 2 and Table 1; N3—H5···N2^i^) forming a dimeric molecular structure, which stabilizes the crystal packing. Symmetry codes: (i) -*x*, -*y* + 1, -*z*.

The crystal structure shows that molecules are additionally connected through intermolecular Br···Br interactions into chains along the crystallographic *b* direction (Fig. 3). The Br···Br distances amount to 3.545 (1) Å, which are shorter than the sum of the van der Waals radii for Br atoms (3.70 Å).

### Experimental

Starting materials were commercially available and were used without further purification. The synthesis of 5-bromosalicylaldehyde thiosemicarbazone was adapted from a procedure reported previously (Joseph *et al.*, 2010). 5-Bromosalicylaldehyde thiosemicarbazone (0.5 mmol) was dissolved in tetrahydrofurane (50 ml) and treated with one KOH pellet. After 30 min stirring under slight warming to 333 K, the solution was filtered and added to a nickel acetate tetrahydrate (0.5 mmol) solution in pyridine (10 ml). The reaction mixture was refluxed for 4 h under continuous stirring and showed a brown-red colour. Brown-red crystals of the complex, suitable for X-ray analysis, were obtained after six weeks by adding a 3:1 mixture of dimethylformamide and toluene (80 ml) to the reaction solution.

### Refinement

H atoms attached to C atoms were positioned with idealized geometry and were refined isotropic with *U*_eq_(H) set to 1.2 times of the *U*_eq_(C) using a riding model with C—H = 0.93 Å. H atoms attached to N atoms atoms were positioned with idealized geometry and were refined isotropically with *U*_eq_(H) set to 1.2 times of *U*_eq_(N) using a riding model with N3—H1 = 0.7822 Å and N3—H2 = 0.8025 Å.

### Crystal data

**Table:**

[Ni(C_8_H_6_BrN_3_OS)(C_5_H_5_N)]	*F*(000) = 816
*M**_r_* = 409.94	*D*_x_ = 1.881 Mg m^−^^3^
Monoclinic, *P*2_1_/*c*	Mo *K*α radiation, λ = 0.71073 Å
Hall symbol: -P 2ybc	Cell parameters from 17227 reflections
*a* = 12.2447 (4) Å	θ = 2.9–27.5°
*b* = 4.1135 (1) Å	µ = 4.25 mm^−^^1^
*c* = 31.1380 (11) Å	*T* = 293 K
β = 112.646 (1)°	Needle, red
*V* = 1447.46 (8) Å^3^	0.93 × 0.10 × 0.05 mm
*Z* = 4	

### Data collection

**Table:**

Nonius KappaCCD diffractometer	3224 independent reflections
Radiation source: fine-focus sealed tube, Bruker Kappa CCD	2697 reflections with *I* > 2σ(*I*)
Graphite monochromator	*R*_int_ = 0.051
Detector resolution: 9 pixels mm^-1^	θ_max_ = 27.6°, θ_min_ = 3.3°
CCD rotation images, thick slices scans	*h* = −15→15
Absorption correction: multi-scan (Blessing, 1995)	*k* = −5→5
*T*_min_ = 0.443, *T*_max_ = 0.830	*l* = −40→40
13946 measured reflections	

### Refinement

**Table:**

Refinement on *F*^2^	Primary atom site location: structure-invariant direct methods
Least-squares matrix: full	Secondary atom site location: difference Fourier map
*R*[*F*^2^ > 2σ(*F*^2^)] = 0.031	Hydrogen site location: inferred from neighbouring sites
*wR*(*F*^2^) = 0.083	H-atom parameters constrained
*S* = 1.05	*w* = 1/[σ^2^(*F*_o_^2^) + (0.0442*P*)^2^ + 0.6555*P*] where *P* = (*F*_o_^2^ + 2*F*_c_^2^)/3
3224 reflections	(Δ/σ)_max_ = 0.001
190 parameters	Δρ_max_ = 0.61 e Å^−^^3^
0 restraints	Δρ_min_ = −0.66 e Å^−^^3^

### Special details

**Table:**

Geometry. All e.s.d.'s (except the e.s.d. in the dihedral angle between two l.s. planes) are estimated using the full covariance matrix. The cell e.s.d.'s are taken into account individually in the estimation of e.s.d.'s in distances, angles and torsion angles; correlations between e.s.d.'s in cell parameters are only used when they are defined by crystal symmetry. An approximate (isotropic) treatment of cell e.s.d.'s is used for estimating e.s.d.'s involving l.s. planes.
Refinement. Refinement of *F*^2^ against ALL reflections. The weighted *R*-factor *wR* and goodness of fit *S* are based on *F*^2^, conventional *R*-factors *R* are based on *F*, with *F* set to zero for negative *F*^2^. The threshold expression of *F*^2^ > σ(*F*^2^) is used only for calculating *R*-factors(gt) *etc*. and is not relevant to the choice of reflections for refinement. *R*-factors based on *F*^2^ are statistically about twice as large as those based on *F*, and *R*- factors based on ALL data will be even larger.

### Fractional atomic coordinates and isotropic or equivalent isotropic displacement parameters (Å^2^)

**Table:**

	*x*	*y*	*z*	*U*_iso_*/*U*_eq_	
Br1	1.37247 (2)	0.72025 (7)	0.228104 (10)	0.04622 (11)	
Ni1	0.80812 (3)	−0.06339 (9)	0.089703 (11)	0.03527 (11)	
S1	0.71884 (6)	−0.35252 (19)	0.02859 (2)	0.04380 (17)	
O1	0.87955 (16)	0.1736 (5)	0.14418 (7)	0.0419 (4)	
N1	0.93997 (18)	−0.0757 (5)	0.07408 (7)	0.0339 (4)	
N2	0.9399 (2)	−0.2507 (6)	0.03540 (8)	0.0403 (5)	
N3	0.8245 (2)	−0.5541 (7)	−0.02746 (9)	0.0519 (6)	
H1	0.8831	−0.6069	−0.03	0.062*	
H2	0.7769	−0.6986	−0.0334	0.062*	
N4	0.67067 (19)	−0.0743 (6)	0.10494 (8)	0.0383 (5)	
C1	1.0684 (2)	0.2490 (6)	0.13889 (9)	0.0350 (5)	
C2	0.9877 (2)	0.2887 (6)	0.16117 (9)	0.0357 (5)	
C3	1.0269 (2)	0.4575 (7)	0.20359 (10)	0.0423 (6)	
H3	0.9751	0.4858	0.2187	0.051*	
C4	1.1399 (2)	0.5829 (7)	0.22359 (10)	0.0428 (6)	
H4	1.1642	0.6923	0.2519	0.051*	
C5	1.2167 (2)	0.5430 (6)	0.20077 (9)	0.0377 (5)	
C6	1.1830 (2)	0.3830 (7)	0.15955 (10)	0.0377 (5)	
H6	1.2358	0.3616	0.1448	0.045*	
C7	1.0394 (2)	0.0704 (7)	0.09658 (9)	0.0380 (6)	
H7	1.0971	0.0579	0.0841	0.046*	
C8	0.8393 (2)	−0.3869 (7)	0.01258 (9)	0.0390 (6)	
C9	0.5660 (2)	0.0532 (7)	0.07772 (10)	0.0439 (6)	
H9	0.558	0.1437	0.0493	0.053*	
C10	0.4699 (3)	0.0547 (9)	0.09040 (12)	0.0553 (8)	
H10	0.3986	0.1475	0.0711	0.066*	
C11	0.4810 (3)	−0.0826 (9)	0.13187 (12)	0.0586 (8)	
H11	0.4172	−0.0845	0.1411	0.07*	
C12	0.5869 (3)	−0.2171 (8)	0.15973 (11)	0.0569 (8)	
H12	0.5955	−0.3131	0.1879	0.068*	
C13	0.6808 (3)	−0.2087 (8)	0.14557 (10)	0.0476 (7)	
H13	0.7528	−0.2985	0.1647	0.057*	

### Atomic displacement parameters (Å^2^)

**Table:**

	*U*^11^	*U*^22^	*U*^33^	*U*^12^	*U*^13^	*U*^23^
Br1	0.03290 (15)	0.0510 (2)	0.05011 (18)	−0.00359 (11)	0.01082 (12)	−0.00312 (13)
Ni1	0.03052 (17)	0.0412 (2)	0.03447 (17)	0.00019 (13)	0.01296 (13)	0.00106 (13)
S1	0.0378 (3)	0.0507 (4)	0.0424 (4)	−0.0046 (3)	0.0148 (3)	−0.0052 (3)
O1	0.0321 (9)	0.0540 (12)	0.0406 (10)	−0.0025 (8)	0.0151 (8)	−0.0049 (9)
N1	0.0352 (10)	0.0337 (11)	0.0336 (10)	0.0034 (8)	0.0141 (9)	0.0010 (9)
N2	0.0419 (12)	0.0419 (13)	0.0399 (12)	0.0010 (10)	0.0189 (10)	−0.0039 (10)
N3	0.0488 (14)	0.0583 (17)	0.0502 (14)	−0.0046 (12)	0.0210 (12)	−0.0152 (12)
N4	0.0346 (11)	0.0443 (13)	0.0356 (11)	−0.0030 (9)	0.0130 (9)	−0.0002 (9)
C1	0.0327 (12)	0.0355 (13)	0.0367 (13)	0.0030 (10)	0.0132 (10)	0.0025 (10)
C2	0.0297 (11)	0.0387 (14)	0.0389 (13)	0.0030 (10)	0.0134 (10)	0.0046 (11)
C3	0.0359 (13)	0.0526 (17)	0.0410 (13)	0.0018 (12)	0.0179 (11)	−0.0023 (12)
C4	0.0387 (13)	0.0475 (16)	0.0393 (13)	0.0025 (12)	0.0118 (11)	−0.0041 (12)
C5	0.0289 (11)	0.0358 (14)	0.0438 (14)	0.0008 (10)	0.0089 (10)	0.0030 (11)
C6	0.0311 (12)	0.0391 (14)	0.0443 (14)	0.0020 (10)	0.0159 (11)	0.0018 (11)
C7	0.0348 (12)	0.0405 (15)	0.0426 (14)	0.0023 (11)	0.0191 (11)	0.0007 (11)
C8	0.0431 (14)	0.0363 (14)	0.0376 (13)	0.0049 (11)	0.0157 (11)	0.0021 (11)
C9	0.0365 (13)	0.0535 (18)	0.0393 (14)	0.0004 (12)	0.0120 (11)	0.0060 (12)
C10	0.0349 (14)	0.072 (2)	0.0588 (18)	0.0048 (14)	0.0179 (13)	0.0068 (16)
C11	0.0478 (17)	0.076 (2)	0.064 (2)	−0.0003 (16)	0.0339 (16)	0.0031 (17)
C12	0.062 (2)	0.071 (2)	0.0460 (17)	−0.0009 (17)	0.0303 (16)	0.0069 (16)
C13	0.0418 (14)	0.0607 (19)	0.0390 (14)	0.0018 (13)	0.0141 (12)	0.0074 (13)

### Geometric parameters (Å, º)

**Table:**

Br1—C5	1.909 (3)	C2—C3	1.403 (4)
Ni1—N1	1.858 (2)	C3—C4	1.380 (4)
Ni1—O1	1.8576 (19)	C3—H3	0.93
Ni1—N4	1.917 (2)	C4—C5	1.390 (4)
Ni1—S1	2.1516 (8)	C4—H4	0.93
S1—C8	1.735 (3)	C5—C6	1.358 (4)
O1—C2	1.311 (3)	C6—H6	0.93
N1—C7	1.295 (3)	C7—H7	0.93
N1—N2	1.403 (3)	C9—C10	1.378 (4)
N2—C8	1.289 (4)	C9—H9	0.93
N3—C8	1.373 (4)	C10—C11	1.367 (5)
N3—H1	0.7822	C10—H10	0.93
N3—H2	0.8025	C11—C12	1.369 (5)
N4—C9	1.341 (4)	C11—H11	0.93
N4—C13	1.342 (4)	C12—C13	1.381 (4)
C1—C6	1.411 (4)	C12—H12	0.93
C1—C2	1.419 (3)	C13—H13	0.93
C1—C7	1.429 (4)		
			
N1—Ni1—O1	95.87 (9)	C5—C4—H4	120.5
N1—Ni1—N4	177.00 (10)	C6—C5—C4	121.4 (2)
O1—Ni1—N4	86.32 (9)	C6—C5—Br1	119.63 (19)
N1—Ni1—S1	87.15 (7)	C4—C5—Br1	118.9 (2)
O1—Ni1—S1	176.46 (6)	C5—C6—C1	120.5 (2)
N4—Ni1—S1	90.60 (7)	C5—C6—H6	119.8
C8—S1—Ni1	95.77 (10)	C1—C6—H6	119.8
C2—O1—Ni1	127.26 (17)	N1—C7—C1	125.8 (2)
C7—N1—N2	113.1 (2)	N1—C7—H7	117.1
C7—N1—Ni1	125.23 (18)	C1—C7—H7	117.1
N2—N1—Ni1	121.70 (16)	N2—C8—N3	118.8 (2)
C8—N2—N1	112.4 (2)	N2—C8—S1	122.9 (2)
C8—N3—H1	115.1	N3—C8—S1	118.2 (2)
C8—N3—H2	114.2	N4—C9—C10	122.3 (3)
H1—N3—H2	112.6	N4—C9—H9	118.8
C9—N4—C13	118.4 (2)	C10—C9—H9	118.8
C9—N4—Ni1	123.42 (18)	C11—C10—C9	118.9 (3)
C13—N4—Ni1	118.17 (19)	C11—C10—H10	120.5
C6—C1—C2	119.3 (2)	C9—C10—H10	120.5
C6—C1—C7	118.2 (2)	C10—C11—C12	119.4 (3)
C2—C1—C7	122.5 (2)	C10—C11—H11	120.3
O1—C2—C3	119.0 (2)	C12—C11—H11	120.3
O1—C2—C1	123.2 (2)	C11—C12—C13	119.3 (3)
C3—C2—C1	117.8 (2)	C11—C12—H12	120.4
C4—C3—C2	122.0 (2)	C13—C12—H12	120.4
C4—C3—H3	119	N4—C13—C12	121.7 (3)
C2—C3—H3	119	N4—C13—H13	119.2
C3—C4—C5	118.9 (3)	C12—C13—H13	119.2
C3—C4—H4	120.5		
			
N1—Ni1—S1—C8	1.82 (11)	C3—C4—C5—C6	−0.3 (4)
N4—Ni1—S1—C8	−176.20 (12)	C3—C4—C5—Br1	179.4 (2)
N1—Ni1—O1—C2	1.9 (2)	C4—C5—C6—C1	−0.7 (4)
N4—Ni1—O1—C2	179.8 (2)	Br1—C5—C6—C1	179.7 (2)
O1—Ni1—N1—C7	−4.3 (2)	C2—C1—C6—C5	1.3 (4)
S1—Ni1—N1—C7	177.6 (2)	C7—C1—C6—C5	−177.2 (2)
O1—Ni1—N1—N2	175.74 (19)	N2—N1—C7—C1	−175.7 (2)
S1—Ni1—N1—N2	−2.40 (18)	Ni1—N1—C7—C1	4.3 (4)
C7—N1—N2—C8	−178.1 (2)	C6—C1—C7—N1	177.7 (3)
Ni1—N1—N2—C8	1.9 (3)	C2—C1—C7—N1	−0.7 (4)
O1—Ni1—N4—C9	119.2 (2)	N1—N2—C8—N3	177.0 (2)
S1—Ni1—N4—C9	−62.5 (2)	N1—N2—C8—S1	0.2 (3)
O1—Ni1—N4—C13	−58.9 (2)	Ni1—S1—C8—N2	−1.6 (3)
S1—Ni1—N4—C13	119.3 (2)	Ni1—S1—C8—N3	−178.4 (2)
Ni1—O1—C2—C3	−178.72 (19)	C13—N4—C9—C10	1.0 (4)
Ni1—O1—C2—C1	0.8 (4)	Ni1—N4—C9—C10	−177.1 (2)
C6—C1—C2—O1	179.6 (2)	N4—C9—C10—C11	−0.9 (5)
C7—C1—C2—O1	−2.0 (4)	C9—C10—C11—C12	0.1 (5)
C6—C1—C2—C3	−0.9 (4)	C10—C11—C12—C13	0.6 (5)
C7—C1—C2—C3	177.5 (2)	C9—N4—C13—C12	−0.2 (4)
O1—C2—C3—C4	179.5 (3)	Ni1—N4—C13—C12	178.0 (2)
C1—C2—C3—C4	0.0 (4)	C11—C12—C13—N4	−0.6 (5)
C2—C3—C4—C5	0.6 (4)		

### Hydrogen-bond geometry (Å, º)

**Table:**

*D*—H···*A*	*D*—H	H···*A*	*D*···*A*	*D*—H···*A*
N3—H1···N2^i^	0.78	2.31	3.095 (3)	178

Symmetry code: (i) −*x*+2, −*y*−1, −*z*.
